# Mesenchymal Stem Cell-Derived Exosomes: Toward Cell-Free Therapeutic Strategies in Chronic Kidney Disease

**DOI:** 10.3389/fmed.2022.816656

**Published:** 2022-03-21

**Authors:** Qinghua Cao, Chunling Huang, Xin-Ming Chen, Carol A. Pollock

**Affiliations:** Renal Medicine, Kolling Institute of Medical Research, Sydney Medical School, University of Sydney, Royal North Shore Hospital, St Leonards, NSW, Australia

**Keywords:** exosome, chronic kidney disease, mesenchymal stem cells, therapy, new advances, regeneration

## Abstract

Chronic kidney disease (CKD) is rising in global prevalence and has become a worldwide public health problem, with adverse outcomes of kidney failure, cardiovascular disease, and premature death. However, current treatments are limited to slowing rather than reversing disease progression or restoring functional nephrons. Hence, innovative strategies aimed at kidney tissue recovery hold promise for CKD therapy. Mesenchymal stem cells (MSCs) are commonly used for regenerative therapy due to their potential for proliferation, differentiation, and immunomodulation. Accumulating evidence suggests that the therapeutic effects of MSCs are largely mediated by paracrine secretion of extracellular vesicles (EVs), predominantly exosomes. MSC-derived exosomes (MSC-Exos) replicate the functions of their originator MSCs via delivery of various genetic and protein cargos to target cells. More recently, MSC-Exos have also been utilized as natural carriers for targeted drug delivery. Therapeutics can be effectively incorporated into exosomes and then delivered to diseased tissue. Thus, MSC-Exos have emerged as a promising cell-free therapy in CKD. In this paper, we describe the characteristics of MSC-Exos and summarize their therapeutic efficacy in preclinical animal models of CKD. We also discuss the potential challenges and strategies in the use of MSC-Exos-based therapies for CKD in the future.

## Introduction

Chronic kidney disease (CKD) is a widespread public health problem, with adverse outcomes of kidney failure, cardiovascular disease, and premature death. CKD is more common than is widely known, affecting approximately 10% of the population worldwide (1). Although the causes of CKD may vary, diabetes and hypertension are still the leading causes (1). Irrespective of the multifactorial etiologies of the initial renal injury, progressive renal fibrosis is common to all forms of CKD (2). Although there have been recent advances in therapeutic strategies for CKD, a significant treatment gap remains. Despite targeted control of diabetes, blood pressure, hyperlipidemia and proteinuria, a large proportion of patients with CKD develop end stage kidney disease (ESKD). Kidney transplantation and dialysis are the only options for the management of ESKD, which results in a significant personal and societal burden (3). Hence innovative therapeutic strategies are urgently needed.

With potent self-renewal capabilities and great potential for differentiation and proliferation, stem cell (SC) therapy has emerged as an option for the preservation of renal function and structural repair in kidney diseases (4). Mounting evidence suggests that SCs exert therapeutic effects mostly by differentiation into tissue-specific cells to replace damaged tissue (5, 6). Amongst different types of SCs, the application of mesenchymal stem cells (MSCs) in treating kidney diseases is widely studied and has been shown to be advantageous over the application of other SCs (7). MSCs are multipotent SCs that differentiate into cells of mesenchymal cell lineages and exert important functions in tissue regeneration and repair by virtue of their wide differentiation capacity as well as anti-inflammatory and immunosuppressive properties (8–10). They can be obtained from virtually any type of tissue (tissue-derived MSCs) including bone marrow (BM), umbilical cord (UC), adipose tissue, dental pulp, amniotic fluid, placenta, Wharton’s jelly (WJ), and organs including kidney, liver, spleen, pancreas, brain, lung, and thymus (11–15). MSCs can also be acquired from cells such as induced pluripotent stem cells (iPSCs)(16). Pluripotent stem cells (PSCs) are cells characterized by the capacity to self-renew and to differentiate into one of the three primary germ cell layers of the early embryo and therefore into specialized cell types (17). There are two types of PSC: embryonic stem cells (ESCs) and iPSCs (18). In 2007, it was reported by Shinya Yamanaka that induced PSCs (iPSCs) could be derived from reprogrammed adult human cells by introducing only a few genes (19). This discovery revolutionized the understanding of cell development and Shinya Yamanaka was thus awarded the Nobel Prize in Physiology or Medicine 2012.

To define MSCs with different origins, minimal criteria required include plastic adherence in standard culture conditions, expression of cluster of differentiation (CD) 105, CD73, and CD90, lack of expression of CD45, CD34, CD14 or CD11b, CD79a or CD19, and human leukocyte antigen-DR isotype (HLA-DR) surface molecules, and differentiation into osteoblasts, adipocytes, and chondroblasts *in vitro* (20). As immunoprivileged cells, MSCs rapidly home to injured kidneys and act through paracrine pathways to promote repair (21). They are reported to prevent and/or reverse kidney fibrosis and improve renal function in both experimental models and human patients (22, 23).

However, there are disadvantages in using cell based MSC therapy. These include the difficulty in maintaining a consistent source of cells with a stable phenotype (24) and in delivering large cells intravenously associated with a hazard of pulmonary microvasculature entrapment (25). Furthermore, MSCs have a risk of tumor formation through vascularization, immune regulation, and facilitating tumor interstitial remodeling (26). These disadvantages have restricted their clinical use. Thus, alternative MSC-based and complication-free therapeutic strategies are needed.

**FIGURE 1:**
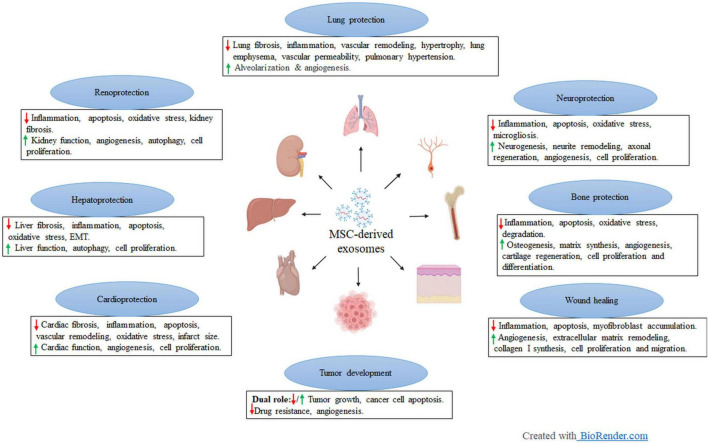
Preclinical application of mesenchymal stem cell-derived exosomes in different disease models. Created with BioRender.com.

Numerous lines of evidence have supported that the therapeutic potential of MSCs is mediated by the secretion of soluble paracrine factors-extracellular vesicles (EVs) including apoptotic bodies (1–5 mm), microvesicles (MVs, 0.1–1 μm), and exosomes (30–150 nm)(27–29). Both MVs and apoptotic bodies are formed by direct budding from the plasma membrane. However, exosomes are produced after the fusion of multivesicular bodies (MVB), which are endocytic organelles containing many luminal vesicles, with the plasma membrane and are characterized by surface expression of CD9, CD63, and CD81 (30, 31). Very recent preclinical studies have identified exosomes as a dominant player in the MSC-mediated repair process of injured tissues (Figure 1). MSC-derived exosomes (MSC-Exos) coordinate intercellular communication and tissue repair through transfer of proteins, RNA, DNA and lipids between cells, which is likely to constitute a novel mode of intercellular communication (32, 33). In this review, we will summarize recent advances regarding the therapeutic application of MSC-Exos in preclinical studies in various experimental CKD models including diabetic kidney disease (DKD), hypertensive CKD and kidney fibrosis, aiming to provide novel insights to the treatment of CKD.

## Exosomes

### Isolation, Identification, and Characterization of Natural Exosomes

Exosomes are small heterogeneous phospholipid-bilayer EVs that can be secreted by almost all type of cells via invagination of the late endosomal membrane (34). Generally, exosomes can now be isolated from conditioned cell culture media or body fluids by differential ultracentrifugation, precipitation, size exclusion chromatography (SEC), filtration, immunoaffinity capture, commercial kits, or microfluidic technologies (35). Each approach has its advantages and disadvantages and there is lack of consensus on a gold standard of isolation. After purification, transmission electron microscopy (TEM) can be used for exosome validation (35). Exosomes contain a wide variety of cytoplasmic or membrane proteins (receptors, enzymes, transcription factors, and ECM components), nucleic acids (mitochondrial DNA, single-stranded DNA, double-stranded DNA, mRNA, and non-coding RNA) and lipids (36, 37). Of note, most exosomes have an evolutionarily conserved set of proteins including tetraspanins (CD81, CD63, and CD9), heat-shock proteins (HSP60, HSP70, and HSP90), ALIX and tumor susceptibility gene 101 (TSG101), which are used as biomarkers to identify exosomes (34).

Naturally, exosomes exhibit the characteristics of their parental cells. Thus, exosomes have been regarded as mini version of the originator cells (34). Emerging evidence has suggested that exosomes are biologically active vesicles regulating physiological and pathological pathways through delivery of functional cargos of proteins, nucleic acids and lipids (34). The cargos of exosomes vary according to the identity and physiological condition of the source cells and the extracellular environment and can be selectively taken up by neighboring or distant cells after the fusion of exosomes to the plasma membrane of recipient cells (38). Once internalized, exosomes fuse with the endosome membrane, followed by the horizontal transfer of their content to the cytoplasm of target cells and modification of their biological activities (39).

### Engineered Exosomes for Drug Delivery

**FIGURE 2:**
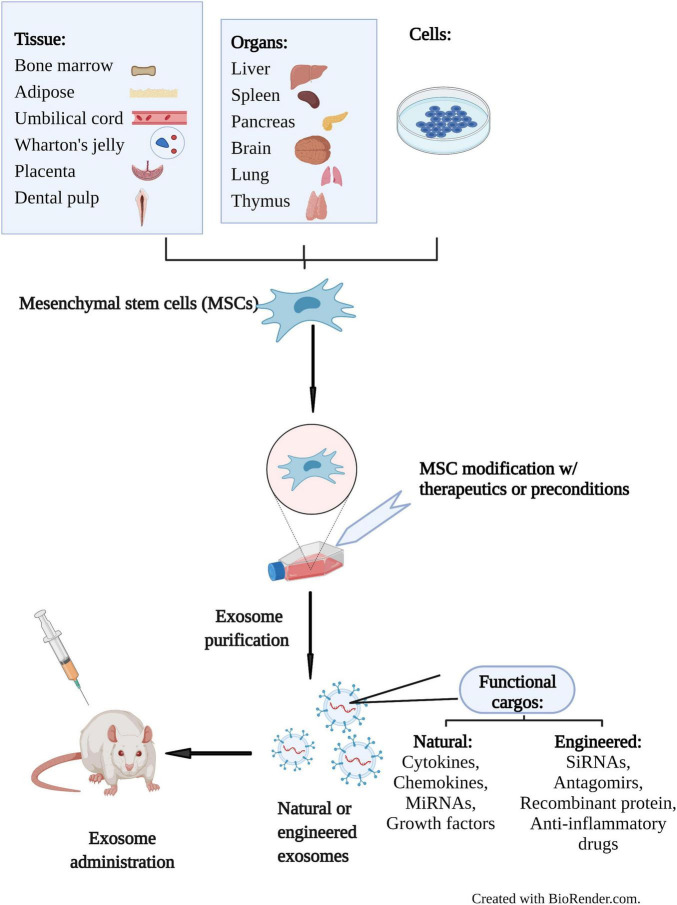
Schematic diagram of therapeutic application of MSC-Exos in preclinical studies. MSCs can be isolated from various sources including tissues, organs, and cells. Exosomes secreted by MSCs can be engineered at the cellular or exosomal level. Natural MSC-Exos exhibit the characteristics of their parental cells through transfer of cargos such as cytokines, chemokines, miRNAs and growth factors. Engineered exosomes can also deliver bioactive siRNAs, antagomirs, recombinant proteins and anti-inflammatory drugs specifically. Administration of MSC-Exos to animal models are used to investigate their therapeutic potential in preclinical studies. Created with BioRender.com.

Recently, natural exosomes have also been engineered as drug carriers to specifically deliver a variety of bioactive molecules, such as short interfering-RNA (siRNA), antagomirs, recombinant proteins, and anti-inflammatory drugs due to their low toxicity, long-term stability, nanoscale size, cargo loading capacity, editable surface and tissue homing capability (40) (Figure 2). The simplest way for cargo loading is to incubate desired cargos with exosome-secreting cells or exosomes to allow diffusion of cargos into exosomes via a concentration gradient (41). Some other strategies include transfection, through which specific plasmids are transduced into cells to ectopically express desired biomolecules in exosomes. In addition to physical treatments (sonication, electroporation, extrusion, freeze-thaw, surfactant treatment and dialysis), *in situ* synthesis have also been applied to generate reconstituted exosomes (42). It is now recognized that natural exosomes spread via free diffusion and are then randomly internalized into recipient cells (43). To achieve specific targeted delivery of reconstituted exosomes, methodologies based on ligand-receptor binding, pH gradient/surface charge, and magnetism have been applied (44).

Compared with synthetic drug carriers, exosomes have several advantages. They can be obtained from patients’ tissues or body fluids with excellent host bio-distribution and biocompatibility, which minimize clearance rate and toxicity (45). For long-distance cell to cell communication, exosomes can also enter the blood and pass through additional biological barriers such as blood-brain barrier to achieve delivery throughout the body (46). Additionally, exosomes can be administrated via different routes (intranasally, intravenously, intraperitoneally, and intracranially), confirming exosome-based drug delivery is highly flexible (40).

Collectively, the utilization of exosomes in therapy has more benefits than their counterpart whole cells. MSCs have shown regenerative potential in the attenuation of kidney injury. Likewise, MSC-Exos represent attractive strategies for the treatment of various kidney diseases including CKD.

## Biochemistry and Functions of MSC-Exos

Mesenchymal stem cell-derived exosomes not only have the advantages of exosomes, but also replicate the biological characteristics of MSCs through transfer of functional cargos, mainly microRNAs (miRNAs) and proteins. MiRNAs are short non-coding RNAs that regulate various physiological cellular processes such as cell death, differentiation, proliferation, metabolism, and pathophysiology of many diseases via regulation of target genes (47, 48). To date, more than 150 miRNAs and over 900 proteins have been identified in cargos of MSC-Exos (49, 50), resulting in the alteration of a variety of activities in target cells via different pathways. The tissue-repairing activities of MSC-Exos involve promoting cell proliferation, dedifferentiation and angiogenesis, whilst simultaneously dampening apoptosis and oxidative stress (51, 52). MiRNA cargos such as miRNA-10a, miRNA-486 were regarded as pro-regenerative miRNAs due to their capability to promote cell proliferation (53, 54) while miRNA-199a-3p was found to downregulate apoptosis-related genes and thereby suppress apoptosis (55, 56). Protein cargos like extracellular matrix metalloproteinase inducer (EMMPRIN) and metalloproteinase-9 (MMP-9) have been reported to stimulate angiogenesis (57, 58). Furthermore, MSC-Exos mitigate inflammatory responses by minimizing infiltration of immune cells such as macrophages, T cells, and NK cells (51). For instance, miRNA-155 (59), miRNA-146a (60), some cytokines such as interleukin-6 (IL-6), IL-10, and growth factor (GFs) hepatocyte growth factor (HGF), contribute to MSC-Exos-mediated immunoregulation (61). Nevertheless, exosomes of different MSC origins contain different biomolecules and thus exhibit heterogeneous characteristics (62, 63).

A comparative proteomic-based analysis through mass spectrometry on the secretome of MSCs revealed that BM-derived MSC (BM-MSCs), adipose-derived MSCs (AD-MSCs), and UC-derived MSC (UC-MSCs) differed in their secretion of anti-oxidative stress or anti-apoptosis molecules involved in central nervous system (CNS) injury (62). Another study by Hoang et al identified differential release of GFs responsible for wound healing by MSC-Exos from BM, adipose and UC (64). Notably, BM-MSC-derived exosomes (BM-MSC-Exos) was superior in inducing primary dermal fibroblasts (64). BM-MSC-Exos have enhanced regeneration capacity by virtue of induction of angiogenesis; AD-MSC-derived exosomes (AD-MSC-Exos) function as major immunomodulators and UC-MSC-derived exosomes (UC-MSC-Exos) mostly participate in tissue repair (63, 65). In spite of the heterogeneity, growing evidence demonstrate that MSC-Exos offer a novel cell-free therapeutic opportunity, as an alternative to MSCs, for treatment of various pathological conditions including neurological disorders, liver or lung damage and acute or chronic kidney injury (50, 66).

## Therapeutic Potential of MSC-Exos for CKD

Mesenchymal stem cells have exhibited promising efficacy in alleviating kidney injury in experimental CKD (67, 68). MSC-Exos possess repair functions similar to MSCs and have been widely used in CKD particularly DKD and kidney fibrosis to overcome the limitations of MSCs. Although the functions of MSC-Exos may vary depending on the cellular source of MSCs, they are in general therapeutic. Among all preclinical studies, heterogenous experiment settings including different doses and schedules, various routes of administration (tail infusion, organ perfusion, or the direct application in the kidney) and distinct CKD animal models were applied.

### MSC-Exos for DKD

Diabetic kidney disease, a microvascular complication of diabetes mellitus (DM), is the most common form of CKD, and is likely to increase in epidemic proportions globally (69). Diabetic patients with kidney disease have a greater mortality risk than those without kidney disease. With the prevalence of DM in the global adult population expected to increase from 8.8% in 2015 to 10.4% in 2040, the impact of DKD is expected to be an increasingly prominent global health issue (69). In DKD, microalbuminuria is an early, although not invariable, clinical manifestation and portends an increased risk for progressive kidney damage. Hyperglycemia activates various inflammatory pathways through direct mechanisms to induce reactive oxygen species, oxidative stress, renin-angiotensin-aldosterone system (RAAS) activation, profibrotic cytokines, including transforming growth factor-beta (TGF-β), and advanced glycation end-products (70). This leads collectively to apoptosis, podocyte and tubular damage and associated albuminuria. The increased matrix protein production and decreased protein degradation leads to deposition of ECM proteins, including collagens and fibronectin (FN) in the glomerular mesangium and tubulointerstitium, resulting in progressive fibrosis (70, 71). To investigate the therapeutic effects of MSC-Exos in DKD, STZ-DKD *in vivo* model (mice or rats) and *in vitro* high glucose (HG)-treated cell lines of podocyte, tubular epithelial cells (TECs) and glomerular endothelial cells were commonly used. The efficacy of MSC-Exos in treating rodent DKD is summarized in Table 1.

**TABLE 1:** Summary of therapeutic effects of MSC-Exos from various sources in preclinical models of DKD.

MSC Source	Model	Dose	Administration	Effects	Mechanism of action	References
Rat bone marrow	*In vivo*: STZ-induced DKD	Single: 5.3 × 10^7^	Renal subcapsular	↓Renal tubule expansion, vacuolation, tubular atrophy ↓Degeneration	↓ TGF- β ZO-1 was maintained ↓Inflammatory cell infiltration	(71)
	*In vitro*: primary TECs	Not stated	Co-culture	↓Degeneration ↓Apoptosis		
Rat bone marrow	STZ-induced DKD	100μg/kg once per day × 4 weeks	Intravenous (Tail vein)	↑Autophagy: ↑ LC3-II/LC-I, p62, Beclin-1 ↓ BUN, Scr, Glu, proteinuria at 10 and 12 weeks ↓ Fibrosis	↓ mTOR, S6K1, p62 ↓ Collagen, FN ↓TGF-β	(77)
Mouse adipose	*In vivo*: spontaneous diabetes	Single: not stated, 12-week therapy	Intravenous (Tail vein)	↓BUN, creatinine, proteinuria ↑ Autophagy ↓ Podocyte apoptosis	↑miR-486, ↓Smad1/mTOR activation ↓Cleaved caspase 3	(79)
	*In vitro*: HG- treated MPC5	25 μg/ml for 48 h	Co-culture	↑Cell viability ↓Apoptosis		
Mouse adipose	*In vitro*: HG- treated MPC5	Not stated	Co-culture	↓Podocyte EMT ↑miR-215-5p, -879-5p, -3066-5p, -7a-5p	↓ ZEB2 transcription	(85)
Adipose	*In vivo*: STZ-induced DKD	50 μg twice weekly × 3	Intravenous (Caudal vein)	↓Glu, Scr, UACR, kidney/body weight ↓Mesangial hyperplasia ↓Kidney fibrosis	Delivery of miR-125a ↓HDAC1, ET-1	(88)
	*IN vitro*: HG-treated rat GMC	Not stated	Co-culture	↓ IL-6, Col-I and FN ↑Bcl-2 and Bax		
Human umbilical cord	*In vitro*: HG-treated HK2, NRK-52E and hrGECs	25, 50, and 100 μg/ml for 24 h	Co-culture	↓TGF-β, IL-6, IL-1β, and TNF-α	Secretion of EGF, FGF, HGF, and VEGF	(73)
Human urine	*In vivo*: STZ-induced DKD	Multiple: 100 μg weekly × 12	Intravenous (Tail vein)	↓Urine volume, albuminuria ↓Apoptosis of podocyte and tubular cells ↑Glomerular endothelial cell proliferation ↑ Angiogenesis	↓Caspase-3 Delivery of VEGF, TGF-β 1, angiogenin, BMP-7	(89)
	*In vitro*: HG- treated immortalized human podocytes	5, 10, and 50 μg/ml for 72 h	Co-culture	↓Podocyte apoptosis		
Human urine	*In vivo*: STZ-induced DKD	100 μg once weekly × 12	Intravenous (Tail vein)	↓Glu, KW, BUN, Scr, Ucr ↓Podocyte injury ↓Apoptosis	↓VEGFA, MCP-1, TGF-β1 and TNF-α. ↓ Bax and Caspase-3	(90)
	*In vitro*: HG- treated human podocytes		Co-culture	↑Cell viability ↓Apoptosis		

*AD-MSCs, adipose-derived mesenchymal stem cells; Bax, Bcl-2-associated X protein; Bcl-2, B-cell lymphoma 2; BMP-7, bone morphogenetic protein-7; BUN, blood urea nitrogen; CK1d, casein kinase 1d; COL-1, collagen-1; DKD, diabetic kidney disease; EMT, epithelial-mesenchymal transition; ET-1, endothelin-1; FGF, fibroblast growth factors; FN, fibronectin; Glu, blood glucose; GMC, glomerular mesangial cell; GSH, glutathione; HDAC1, histone deacetylase 1; HG, high glucose; HGF, hepatocyte growth factor; IL-6, interleukin-6; LC3, microtubule-associated protein light chain 3; miR, microRNA; KW, kidney weight; MCP-1, monocyte chemoattractant protein-1; MSC, mesenchymal stem cells; mTOR, mammalian target of rapamycin; ROS, reactive oxygen species; S6K1, ribosomal protein S6 kinase beta-1; Scr, serum creatinine; STZ, streptozotocin; TGF-β, transforming growth factor-β; TECs, tubular epithelial cells; TGF-βR1, transforming growth factor-β type 1 receptor; TNF-α, tumor necrosis factor-α; Ucr, urine creatinine; VEGF, vascular endothelial growth factor; ZO-1, tight junction protein 1; 2K-1C, 2 kidneys, 1 clip model.*

#### Involvement of MSC-Exos From Tissues in DKD

The various cargos including GFs and therapeutic miRNAs delivered by MSC-Exos exert significant effects on restoring renal function, enhancing autophagy, attenuating podocyte injury and mitigating kidney fibrosis. MSCs originate from a wide range of sources and were first discovered from BM (72). In the study by Nagaishi et al., exosomes derived from BM-MSCs were delivered to the subcapsular region of the kidney in STZ-induced diabetic rats *in vivo* (73). These exosomes ameliorated kidney injury, inflammatory cell infiltration and TGF-β production as well as maintained the expression of tight junction protein-1 (ZO-1). Consistently, BM-MSC-Exos also suppressed apoptosis and degeneration of primary TECs from STZ-induced diabetic rats *in vitro* (73). UC, a conduit between the placenta and the developing embryo, is another popular source of MSCs due to the easy, safe and non-invasive way of collection, low immunogenicity, and high paracrine potential (74). *In vitro*, UC-MSC-Exos dramatically downregulated HG-induced pro-inflammatory cytokines including TGF-β, IL-6, IL-1β, and TNF-α in both renal TEC cell lines (NRK-52E, HK2) and human renal glomerular endothelial cell line (hrGECs) via their horizontal transfer of large amounts of GFs including epidermal growth factor (EGF), fibroblast growth factor (FGF), HGF and vascular endothelial growth factor (VEGF) (75).

Autophagy is an intracellular lysosome-dependent degradative process, which maintains cellular homeostasis and integrity through removing damaged macromolecules and organelles (76). Additionally, autophagy is critical to provide energy and molecular building blocks by recycling macromolecules in response to nutrient and environmental stress (77). Impairment of autophagy in renal cells in patients with DM contributes to the progression of DKD via mammalian target of rapamycin (mTOR) pathway activation (78). Studies have proven that MSC-Exos can effectively restore autophagy activity by decreasing mTOR. Rats administered BM-MSC-Exos revealed significantly enhanced autophagy markers microtubule-associated protein light chain 3 (LC3)-II/LC3-I and p62 protein expression compared to the animals with DKD (79). Consistently, fibrotic markers including TGF-β and FN were also inhibited, suggesting a potent anti-fibrotic effect of MSC-Exos. The protective impact of MSC-Exos can be blocked by autophagy inhibitors including 3-methyladenine (3-MA) and chloroquine in rats, confirming the involvement of autophagy in the MSC-Exos mediated renoprotection (79).

Adipose-derived stem cells are also MSCs obtained from adipose tissue (80). Unlike BM-MSCs, AD-MSCs can be obtained by a minimally invasive procedure and thus are also promising for tissue regeneration. AD-MSC-Exos enhanced autophagy and reduced podocyte apoptosis, leading to attenuated DKD as evidenced by reduced levels of urine protein, serum creatinine (Scr) and blood urea nitrogen (BUN) in mice. Consistently, AD-MSC-Exos reversed autophagy downregulation and suppressed podocyte apoptosis *in vitro* (81).

#### MiRNAs as Major Bioactive Cargos of MSC-Exos

MicroRNAs have been found to be packed and protected from proteases and RNAses in EVs (82, 83) and are the most abundant content in human plasma derived exosomal RNAs (84). Investigations on mechanisms by which MSC-Exos elicit their renoprotection verify that apart from proteins such as various GFs, certain miRNAs are the main contents of exosomes contributing to their regenerative potential (32). In HG-stimulated podocyte *in vitro*, miRNA-486 from AD-MSC-Exos inhibited Smad1 and mTOR activation, leading to increased autophagy and reduced podocyte apoptosis (81). These beneficial effects can be neutralized in the presence of miRNA-486 inhibitor, further supporting that AD-MSC-Exos promoted survival of podocytes through miRNA-dependent mechanisms.

In most CKD, the breakdown of the glomerular filtration barrier (GFB) manifests as proteinuria and is subsequently associated with loss of normal kidney function (85). Podocytes, which are specialized visceral epithelial cells, are an independent component of the GFB. They plays an essential role in maintaining the integrity of GFB (85). HG induces epithelial-mesenchymal transition (EMT) and may initiate podocyte injury, resulting in GFB destruction (86). Jin et al found that AD-MSC-Exos administration mitigated HG-induced podocyte EMT due to the restoration of miRNAs including miRNA-215-5p, miRNA-879-5p, miRNA-3066-5p, and miRNA-7a-5p (87). As miRNA-215-5p mimics abrogated HG-induced EMT in podocytes and miRNA-215-5p inhibitors counteracted the protective effect of the AD-MSC-Exos, miRNA-251-5p is regarded as a main player facilitating protection of AD-MSC-Exos on podocyte damage (87). Histone deacetylase 1 (HDAC1)/endothelin-1 (ET-1) axis upregulation was observed in DKD rats and HG-stimulated glomerular mesangial cells (GMCs). High ET-1‘expression induces insulin resistance and increases glomerular permeability, thereby promoting the progression of DKD (88, 89). A recent study suggested AD-MSC-Exos alleviated DKD through delivering miRNA-125a, which targeted the HDAC1/ET1 axis directly to block inflammation and fibrosis (90).

#### Involvement of Exosomes From Urine-Derived Stem Cells in DKD

Urine-derived stem cells (USCs) display classical features of MSCs. Importantly, they can be isolated from urine with a cheap and non-invasive procedure, whereas most adult SCs require invasive procedures (91). Moreover, USCs can differentiate into renal cells, therefore representing huge benefits for application in the treatment of kidney diseases (92, 93). Intravenous injections of USCs-derived exosomes (USC-Exos) alleviated albuminuria in diabetic rats through inhibiting podocytic apoptosis and increasing glomerular endothelial cell proliferation and mesangial angiogenesis in the early stage of DKD (94). The horizontal transfer of podocyte survival factor (bone morphogenetic protein-7, BMP-7) and proangiogenic factors (VEGF, TGF-β, and angiogenin) from USCs-Exos to resident cells mediated nephroprotection by USC-Exos (94). Another study on USC-Exos showed that USC-Exos delivered miRNA-16-5p to the injured kidney and mitigated renal functional impairment (decreased BUN, Scr, and Ucr) in the STZ-DKD rat model (95). The mechanism of renoprotection was attributed to the downregulation of VEGFA, monocyte chemoattracting protein-1 (MCP-1), TGF-β1, TNF-α, and apoptosis-associated protein including B-cell lymphoma-2 (Bcl-2), Bcl-2-associated X protein (Bax) and Caspase-3. *In vitro*, USC-Exos enriched with miRNA-16-5p also led to inhibition of VEGF and offered protection against HG-induced podocyte apoptosis (95).

### MSC-Exos for Hypertensive CKD

Hypertension, a complex multifactorial disease, is also one of the leading causes of CKD due to the deleterious effects of increased blood pressure (BP) on the kidney. Chronic hypertension leads to changes in the systemic and renal macro and microvasculature, resulting in loss of renal auto-regulation, increased glomerular capillary pressure and hyperfiltration-mediated tubular injury (96). Hyperfiltration contributes to glomerular proteinuria, which promotes the release of inflammatory cytokines and GFs by GMCs and TECs (97). In addition, hypertension induces vascular stretch, endothelial dysfunction and the consequent activation of the intra-renal renin-angiotensin system (RAS), which amplifies the release of cytokines and GFs, recruitment of inflammatory cells, increased ECM production and finally progressive glomerular and tubulointerstitial fibrosis (98). Patients with diabetes commonly have hypertension due to chronic hyperglycemia-induced dysfunction of the vasculature (99).

Studies investigating the use of MSC-Exos as therapeutic agents in hypertension-associated CKD are scant. Aliotta et al found that exosomes isolated from both human and murine MSCs were effective in reversing pulmonary hypertension in a mouse model. The beneficial effects may be mediated by cargos of anti-inflammatory and anti-proliferative miRNAs (miRNAs-34a, -122, -124, and -127) that dampen angiogenesis, blunt neoplastic cell proliferation and elicit senescence of vascular smooth muscle cells (SMCs) and endothelial progenitor cells (EPCs) (100). Lindoso et al reported that multiple injections of EVs isolated from adipose-MSCs protected the kidney from hypertensive damage by downregulating the pro-inflammatory molecules MCP-1 and plasminogen activating inhibitor-1 (PAI-1) and reducing macrophage recruitment to the kidney in a hypertensive rat model. Furthermore, the miRNA-200-TGF-β axis was found to be significantly altered after EV administration, thereby reprogramming EMT signaling and preventing renal inflammation and fibrosis (101).

### MSC-Exos for Kidney Fibrosis

Kidney fibrosis, and in particular tubulointerstitial fibrosis, is the final common outcome of nearly all forms of progressive CKD (2). The histopathology of tubulointerstitial fibrosis is characterized by the deposition of ECM in the interstitium associated with inflammatory cell infiltration, tubular cell damage, fibroblast activation and expansion and rarefaction of the peritubular microvasculature (2). Many studies have established TGF-β as a major profibrotic factor through various mechanisms (102). Once renal fibrosis supervenes, progressive functional decline occurs, which relentlessly progresses leading to dialysis or renal transplantation.

In recent years, apart from diabetic and hypertensive CKD models, several other rodent models of CKD such as unilateral ureteral obstruction (UUO), ischemia-reperfusion injury (IRI) and 2 kidney, 1 clip (2K-1C) unilateral renal artery stenosis model have also been utilized to assess the anti-fibrotic efficacy of MSC-Exos (Table 2). UUO induces severe renal injury, characterized by reduced renal blood flow and glomerular filtration rate within 24 h, followed by interstitial inflammation (peak at 2–3 days), tubular dilation, tubular atrophy and fibrosis within a week. It develops interstitial infiltration of macrophages, tubular cell death, the phenotypic transition of resident renal cells and severe interstitial renal fibrosis with excessive ECM accumulation (103). Renal IRI is one of the leading causes of acute kidney injury (AKI), which temporarily suspends the oxygen and nutrient supply to kidney, inducing robust cellular and molecular responses primarily in TECs. After IRI, the acutely damaged kidney experiences a transition from an unresolved self-healing process to maladaptive repair, resulting in incomplete recovery and progression to kidney fibrosis (104, 105). In the 2K-1C model, one renal artery is constricted to chronically reduce renal perfusion, leading to renal hypertension, hypoxia, activation of RAAS and irreversible renal impairment (106, 107).

#### Natural MSC-Exos

It is now well recognized that the alleviation of the inciting cause of fibrosis alone is not sufficient to restore kidney function as functional nephron tissue is damaged or lost after kidney injury (108, 109). Consequently, MSC-Exos with potential for kidney regeneration might represent an innovative strategy for kidney fibrosis alleviation. Although MSCs have been proven to be derived from virtually all tissues’ adventitial progenitor cells and pericytes, UC-derived MSCs are considered one of the major MSCs sources for clinical and research applications (74). During the prenatal phase, the UC is genetically and physiologically part of the fetus and usually contains two arteries and one vein. These blood vessels are enveloped by mucous connective tissue WJ, which is derived from the extraembryonic mesoderm and exerts a protective function (13). Both UC and WJ are considered as promising sites for MSC collection (13, 74).

A notable mechanism of kidney fibrosis is injury-induced oxidative stress, which is caused by over-production of reactive oxygen species (ROS) that exceed its scavenging capacity (110). Excessive ROS repress the antioxidant enzymes and results in breakdown of cells through lipid peroxidation, DNA fragmentation and protein damage. In addition, ROS can promote the progression of renal interstitial fibrosis by regulating the infiltration of inflammatory cells such as monocytes and macrophages (111). UUO-induced renal damage is associated with oxidative stress-induced renal tubular apoptosis (112). UC-MSC-Exos administered after UUO alleviated kidney fibrosis and restored renal function (decreased BUN and Scr) through inhibition of apoptosis, malondialdehyde (MDA), ROS, and ROS-mediated P38MAPK/ERK signaling pathways. *In vitro*, similar anti-fibrotic effects were also observed in TGF-β1 treated NRK-52E cells (113). Another study investigating the anti-fibrotic effects of UC-MSC-Exos confirmed the involvement of Hippo and yes associated protein (YAP) signaling, which regulates TGF-β-Smad signaling, podocyte mesenchymal-epithelial trans-differentiation and ECM protein synthesis (114). Once the Hippo pathway is activated, it limits tissue growth and cell proliferation through the degradation of YAP. UC-MSC-Exos deliver two major ubiquitination related enzyme casein kinase 1d (CK1d) and E3 ubiquitin ligase-transducin repeats-containing protein (b-TRCP) to trigger ubiquitination and degradation of YAP in TECs. This reduced ECM deposition and attenuated fibrosis associated with UUO. Knockdown of CK1d and b-TRCP abrogated the repairing effects of UC-MSC-Exos on renal fibrosis, implying that the efficacy of UC-MSC-Exos relies on the transportation of these active proteins (114).

**TABLE 2:** Summary of anti-fibrotic effects of MSC-Exos from various sources in preclinical models of kidney fibrosis.

MSC Source	Model	Dose	Administration	Effects	Mechanism of action	References
Human umbilical cord	*In vivo*: UUO	Single: 200μg	Left renal artery	↑Renal function (↓Scr, BUN) ↓Tubular injury ↓ Tubulointerstitial fibrosis ↓ Apoptosis ↑proliferation ↓Oxidative stress	↓ ROS-mediated p38 MAPK/ERK signaling pathway ↓Bax, cleaved caspase-3 ↓ROS, MDA ↑ anti-oxidants: GSH	(111)
	*In vitro*: NRK52E incubated with TGF-β	Not stated	Co-incubation with isolated exosome	↓ Apoptosis ↑Proliferation ↓Oxidative stress		
Human bone marrow	*In vivo*: UUO	Single: released from 1 × 10^6^ MSCs	Intravenous	Exosomes home to injured kidneys ↓Fibrosis	Delivery of miR-let7c ↓Collagen, MMP-9, α-SMA, TGF-βR1	(117)
	*In vitro*: NRK52E incubated with TGF-β	Not stated	Co-incubation with isolated exosome	↓Fibrosis		
Human bone Marrow (Transfected with anti-let-7i-5p)	*In vivo*: UUO	Single: 1 mg/kg	Intravenous	↑Renal function (↓BUN, ↓Scr, ↓Ucr, ↑eGFR) ↓ Fibrosis	↓Let-7i-5p ↓Collagen, FN, α-SMA, ↑TSC1 ↓Phosphorylation of mTORC1, p70S6K and 4E-BP1	(118)
	*In vitro*: NRK52E incubated with TGF-β	Not stated	MSC on Transwell with NRK52E grown on the lower chamber	↓TGF-β1-induced fibrogenic responses ↓EMT		
Human umbilical cord	*In vivo*: UUO	Single: 200 μg	Intravenous	↓Tubulointerstitial fibrosis	Exosomes delivered CK1 1δ and β-TRCP to degrade YAP	(112)
Adipose (Transfected with GDNF)	*In vivo*: UUO	Single: 200 μg	Caudal vein	↓ PTC rarefaction ↓ Tubulointerstitial fibrosis ↑ Endothelial function, angiogenesis	↑SIRT1/p-eNOS ↓α-SMA ↑ VEGF, ↓ HIF-1α	(121)
	*In vitro*: HUVEC against H/SD	Single: 100 μg/ml	Co-incubation with isolated exosome	↓ HUVEC injury ↓Apoptosis ↑Endothelial angiogenesis		
Adipose	*In vivo*: IRI	Single: 100 μg	Caudal vein	↑ Tubular proliferation, regeneration ↓ Interstitial fibrosis ↓Inflammation	↑Sox9 ↓α-SMA, PDGFR- β	(114)
	*In vitro*: primary TECs stimulated with TGF-β	Not stated	Co-incubation with isolated exosome	↓ TGF- β1-induced transformation of TECs to pro-fibrotic phenotype		
Adipose	2K-1C Unilateral renal artery stenosis	Single: 100 μg	Caudal vein	↓HIF-1α Stabilised systolic blood pressure ↑ Natriuresis ↓ Fibrosis ↓ Inflammation	↓Collagen, TGF- β ↑ IL-10	(115)
Pluripotent stem cell	*In vivo*: UUO	Single: 10^11^ particles/ml	Tail vein	↓ Fibrosis ↓ Inflammation	↑SIRT6 ↓β-catenin	(126)
	*In vitro*: NRK-52E	10^6^/10^7^/10^8^ particles/ml	Co-incubation with isolated exosome	↓Col-1, α-SMA ↑E-cadherin		

*AD-MSCs, adipose-derived mesenchymal stem cells; Bax, Bcl-2-associated X protein; Bcl-2, B-cell lymphoma-2; BMP-7, bone morphogenetic protein-7; BUN, blood urea nitrogen; CK1d, casein kinase 1d; COL-1, collagen-1; DKD, diabetic kidney disease; EMT, epithelial-mesenchymal transition; ET-1, endothelin-1; FGF, fibroblast growth factors; FN, fibronectin; GDNF, glial-derived neurotrophic factor; GMC, glomerular mesangial cell; GSH, glutathione; HDAC1, histone deacetylase 1; HG, high glucose; HGF, hepatocyte growth factor; H/SD, hypoxia/serum deprivation; HUVECs, human umbilical vein endothelial cells; IL-6, interleukin-6; IRI, ischemia/reperfusion injury; LC3, microtubule-associated protein light chain 3; miR, microRNA; KW, kidney weight; MCP-1, monocyte chemoattractant protein-1; MDA, malondialdehyde; MSC, mesenchymal stem cells; mTOR, mammalian target of rapamycin; mTORC1, mammalian target of rapamycin complex 1; PDGFR-β, platelet derived growth factor receptor beta; ROS, reactive oxygen species; S6K1, ribosomal protein S6 kinase beta-1; Scr, serum creatinine; SIRT1, sirtuin-1; Sox9, SRY-box transcription factor 9; STZ, streptozotocin; TECs, tubular epithelial cells; TGF-β, transforming growth factor-β; TGF-βR1, transforming growth factor-β type 1 receptor; TNF-α, tumor necrosis factor-α; Ucr, urine creatinine; VEGF, vascular endothelial growth factor; ZO-1, tight junction protein-1; 2K-1C, 2 kidneys, 1 clip model; YAP, Yes-associated protein; UUO, unilateral ureteral obstruction.*

Sex-determining region Y-box transcription factor 9 (Sox-9) is a transcription factor of the sex-determining region Y (SRY) box family and may repair injured kidney (115). AD-MSCs-Exos upregulated Sox9 and prevented TGF-β1-induced transformation of TECs into a pro-fibrotic phenotype *in vitro*. Moreover, AD-MSC-EVs were capable of attenuating kidney fibrosis through improving kidney hypoxia, reducing inflammatory cell infiltration and inflammatory cytokine secretion, and inhibiting the TGF-β1/Smad 3 signaling pathway in mice subjected to unilateral IRI (116). IRI also occurs in donation after circulatory death (DCD) kidneys. To evaluate the renoprotective effect of MSC-EVs on isolated DCD kidney, MSC-EVs were applied as part of the hypothermic machine perfusion (HMP) procedure in a rat DCD model. The addition of MSC-EVs during HMP attenuated the ischemic kidney injury through maintaining the enzymatic machinery critical for cell survival and reduced the reperfusion damage to kidney (117). Beneficial properties of AD-MSC-Exos were also reported in the 2K-1C (118), a renal artery stenosis model. Administration of AD-MSC-Exos were demonstrated to stabilize the systolic blood pressure (SBP), downregulate hypoxia marker HIF-1a and reduce profibrotic gene collogen and TGF-β expression, thus mitigating kidney fibrosis (118). Interestingly, the treatments with AD-MSC-Exos, AD-MSC or AD-MSC-EVs were equally effective in reducing the expression of the fibrotic markers collagen-1 (COL-1) and TGF-β. However, AD-MSCs were the most effective in elevating the expression of the anti-inflammatory IL-10. These difference may be ascribed to the various cargos released and/or to the ability of the vesicles to reach the damaged tissue, which requires further investigation (118). WJ-derived MSCs (WJ-MSCs) are more immune-privileged and exhibit greater immunosuppressive properties compared to BM-MSCs or AD-MSCs. WJ-MSCs mitigated kidney fibrosis triggered by IRI through downregulating HGF versus TGF-β1 expression (119).

#### Engineered MSC-Exos

As mentioned earlier, aside from paracrine transferring their natural biological cargos, exosomes including MSC-Exos can also be engineered to carry different biomolecules to various therapeutic targets. The engineered exosomes have a higher therapeutic potential and efficacy and more specific targeting when compared with naive exosomes (40).

Numerous studies have validated that the anti-fibrotic effect of MSC-Exos can be mediated through the transfer of miRNAs such as miRNA-let7c, which targets fibrosis-associated genes. To deliver miRNA-let7c, Wang et al., utilized lentiviral transduction to construct the engineered human BM-MSCs overexpressing miRNA-let7c. The exosomes released from engineered MSC mediated the transfer of miRNA-let7 to diseased kidney and attenuated UUO-induced kidney fibrosis through repression of fibrotic gene collagen-4 (COL-4), MMP-9, alpha-smooth muscle actin (α-SMA), TGF-β1 and its receptor (120). In another study by Jin et al., exosome-secreting MSCs were transfected with let-7i-5p antagomir (anti-let-7i-5p), and then exosomes were isolated from the transfected MSCs to deliver anti-let-7i-5p oligonucleotides to inhibit the level of let-7i-5p. These engineered exosomes reduced the level of let-7i-5p via delivery of anti-let-7i-5p, reduced ECM deposition and attenuated EMT process in TGF-β1-stimulated NRK-52E cells and in the damaged kidneys of UUO mice, thereby attenuating kidney fibrosis (121). Glial-derived neurotrophic factor (GDNF), an effective neurotrophic factor that protects nigral dopaminergic neurons, promoted the therapeutic effect of MSCs (122, 123). Chen et al transfected GDNF into human AD-MSCs via lentiviral transfection and then exosomes (GDNF-AD-MSC-Exos) were collected from those engineered MSCs. Application of the GDNF-AD-MSC-Exos led to the amelioration of kidney fibrosis in mice with UUO, which was mediated by enhancing SIRT1 signaling and its downstream target, phosphorylated endothelial nitric oxide synthase (p-eNOS), which activated endothelial function and angiogenesis and reduced peritubular capillary loss (124).

### iPSC-Derived MSC-Exos for CKD

All MSC mentioned above are from tissues. Despite promising therapeutic effects, tissue-derived MSCs have been reported to have several weaknesses, such as limited potential to proliferate, difficult to standardize, loss of differentiation capacity, and decreased regenerative efficacy with expansion (125). As mentioned earlier, MSCs can also be produced from cells such as iPSCs. Those single cell-derived MSCs have the characteristics of both MSCs and PSCs and are capable of expanding with high efficiency (17). iPSC-MSCs revealed comparable effects in renoprotection, such as reducing apoptosis and enhancing vascularization (4). EVs directly isolated from iPSC rescued rats from IRI through maintaining functional mitochondria and inhibiting oxidative stress-relevant genes (126). Sirtuin 6 (Sirt6) is an NAD-dependent deacetylase of the Sirtuin family that has been suggested to effectively reverse the fibrotic process in many organs (127, 128). More recently, a study by Liu et al established that intravenous infusion of human iPSC-derived MSC-Exos (iPSC-MSC-Exos) mitigated kidney fibrosis, reduced inflammatory responses, and improved renal function in mice subjected to UUO (129). These anti-fibrotic effects of iPSC-MSC-Exos are mediated through increasing SIRT6 while decreasing β-catenin and its downstream products (PAI-1, Fsp1 and Axin2), elucidating a novel mechanism of MSC-Exos in nephroprotection (129).

### MSC-Exos in Lupus Nephritis

Systemic lupus erythematosus (SLE) is a common autoimmune disease. It is characterized by multi-organ damage resulting from abnormal activation of autoreactive T cells, the presence of pathogenic autoantibodies and deposition of immune complexes (130). LN is the most common and severe organ injury in SLE (131). Over the past decades, there has been several publications investigating the therapeutic application of MSCs in LN in both animal models and humans. BM-MSCs alleviated LN and improved mice survival rate by effectively inhibiting IL-21 production and follicular helper T cell differentiation (132). The combination of MSCs with prednisone or mycophenolate mofetil (MMF) improved survival, reduced the secretion of autoantibody and inflammatory cytokines, and decreased the infiltration of inflammatory cells in the kidney in a mouse model of lupus nephritis (133). In LN patients, allogeneic MSC transplantation (MSCT) resulted in an increased glomerular filtration rate (GFR) and renal remission over 12 months, confirming its therapeutic potential for LN (134). It has been well established that MSC-Exos exert immunomodulatory effects through delivery of immunosuppressive molecules that inhibit infiltration, proliferation, differentiation and activation of immune cells or induce anti-inflammatory cells (135). Additionally, MSC-Exos promote the chemotaxis of anti-inflammatory non-coding RNAs to accelerate tissue healing (136). However, despite the efficacy and clinical potential for therapeutic application in inflammatory glomerular disease indicated by these studies, there are few publications applying MSC-Exos directly in LN animal models or in human patients. Recently, Wei et al reported that miR-20a-containing exosomes are responsible for the alleviation of LN in the mouse lupus model through enhancing autophagy (137). In another study by Chen et al., UC-MSC-Exos attenuated SLE-associated diffuse alveolar hemorrhage (DAH) by regulating macrophage polarization in murine lupus (138). In summary, further studies are warranted for a better understanding of the application of MSC-Exos-based therapy in LN and more generally in glomerular disease.

## Conclusion

Chronic kidney disease is a world-wide pandemic, and its prevalence is rising annually. MSC-Exos transfer a variety of growth factors and non-coding miRNAs to injured renal cells, which attenuate kidney injury and restore kidney function through promoting proliferation, autophagy and angiogenesis, and suppressing inflammation, oxidative stress, apoptosis, EMT, and tubulointerstitial fibrosis. Thus, MSC-Exos represent a novel cell-free therapeutic strategy for the treatment of CKD.

Despite advances in understanding the therapeutic capacity of MSC-Exos in CKD, major issues surrounding large-scale production and purification must be overcome before translation of MSC-Exos therapy to clinical application occurs. The amount of MSC-Exos required for clinical application is high. Recently, new technologies such as 3D culture conditions using hydrogels, spheroid or hollow fibers and bioreactors have been introduced to allow large-scale production of exosomes (139). To optimize the procedures of isolation/purification of exosomes, new approaches such as tangential flow filtration (TFF) and asymmetrical field-flow fractionation (AsFFF) have been applied (140, 141). However, there is a lack of standard techniques to quickly isolate, purify, quantitate, and identify exosomes. Moreover, it still requires further investigation to fully understand the biodistribution and clearance of MSC-Exos upon administration. Biodistribution of systemically administered exosomes is a dynamic process. Although several *in vivo* tracking strategies have been employed, current knowledge of the biodistribution of MSC-Exos is limited.

To conclude, advances in MSC-Exos studies hold a great promise for the regenerative treatment of CKD. Future studies focusing on the standardization of MSC-Exos production, purification, and characterization to improve quality and safety will enable the translation of MSC-Exos into the clinic as efficient therapeutics for CKD.

## Author Contributions

QC conceived and wrote the manuscript. X-MC and CH reviewed the manuscript. CP revised and reviewed the manuscript. All authors have read and agreed to the published version of the manuscript.

## Conflict of Interest

The authors declare that the research was conducted in the absence of any commercial or financial relationships that could be construed as a potential conflict of interest.

## Publisher’s Note

All claims expressed in this article are solely those of the authors and do not necessarily represent those of their affiliated organizations, or those of the publisher, the editors and the reviewers. Any product that may be evaluated in this article, or claim that may be made by its manufacturer, is not guaranteed or endorsed by the publisher.

## Abbreviations

AD-MSCs: adipose-derived mesenchymal stem cells
AD-MSC-Exos: AD-MSC-derived exosomes
AKI: acute kidney injury
AsFFF: asymmetrical field-flow fractionation
α -SMA: α -smooth muscle actin
Bax: Bcl-2-associated X protein
Bcl-2: B-cell lymphoma-2
BM: bone marrow
BM-MSC: bone marrow-derived mesenchymal stem cell
BM-MSC-Exos: BM-MSC-derived exosomes
BMP-7: bone morphogenetic protein-7
b-TRCP: E3 ubiquitin ligase-transducin repeats-containing protein
BUN: blood urea nitrogen
CD: cluster of differentiation
CNS: central nervous system
CK1d: casein kinase 1d
CKD: chronic kidney disease
COL-1: collagen-1
COL-4: collagen-4
DKD: diabetic kidney disease
EGF: epidermal growth factor
EMMPRIN: extracellular matrix metalloproteinase inducer
EMT: epithelial-mesenchymal transition
EPC: endothelial progenitor cells
ESC: embryonic stem cells
ESKD: end stage kidney disease
ET-1: endothelin-1
EV: extracellular vesicles
FGF: fibroblast growth factor
FN: fibronectin
GDNF: glial-derived neurotrophic factor
GF: growth factor
GFB: glomerular filtration barrier
GMCs: glomerular mesangial cells
HDAC1: histone deacetylase 1
HGF: hepatocyte growth factor
HLA-DR: human leukocyte antigen-DR isotype
HSP: heat-shock proteins
IL-6: interleukin-6
iPSC: induced pluripotent stem cells
iPSC-MSC-Exos: iPSC-MSC-derived Exosomes
IRI: ischemia-reperfusion injury
LN: lupus nephritis
MCP-1: monocyte chemoattracting protein-1
MDA: malondialdehyde
MMF: mycophenolate mofetil
MiRNAs: microRNAs
MMP-9: metalloproteinase-9
MSC: mesenchymal stem cells
MSC-Exos: MSC-derived exosomes
MSCT: MSC transplantation
mTOR: mammalian target of rapamycin
MVB: multivesicular bodies
PAI-1: plasminogen activating inhibitor-1
P-eNOS: phosphorylated endothelial nitric oxide synthase
PSC: pluripotent stem cells
RAAS: renin-angiotensin-aldosterone system
ROS: reactive oxygen species
SBP: systolic blood pressure
SC: stem cell
Scr: serum creatinine
SMC: smooth muscle cell
SEC: size exclusion chromatography
SiRNA: short interfering-RNA
Sirt6: sirtuin 6
SLE: systemic lupus erythematosus
Sox9: SRY-box transcription factor 9
SRY: sex-determining region Y
TECs: tubular epithelial cells
TEM: transmission electron microscopy
TFF: tangential flow filtration
TGF- β: transforming growth factor-beta
TSG101: tumor susceptibility gene 101
UC: umbilical cord
USC: urine-derived stem cell
UC-MSC: umbilical cord-derived mesenchymal stem cell
UC-MSC-Exos: UC-MSC-derived exosomes
UUO: unilateral ureteral obstruction
VEGF: vascular endothelial growth factor
WJ: Wharton’s jelly
WJ-MSC: WJ-derived MSC
YAP: yes associated protein
2K-1C, 2 kidney: 1 clip
3-MA: 3-methyladenine.
